# Precision enhanced alignment bonding technique with sacrificial strategy

**DOI:** 10.3389/fbioe.2023.1105154

**Published:** 2023-02-16

**Authors:** Qian Li, Zi Ye, Mingyang Liu, Wei Liu, Pan Zhang, Xiao Sun, Huimin Zhang, Zhenming Li, Lin Gui

**Affiliations:** ^1^ CAS Key Laboratory of Cryogenics, Technical Institute of Physics and Chemistry, Chinese Academy of Sciences, Beijing, China; ^2^ School of Engineering Science, University of Chinese Academy of Sciences, Beijing, China; ^3^ Energy Storage and Novel Technology of Electrical Engineering Department, China Electric Power Research Institute, Beijing, China; ^4^ School of Future Technology, University of Chinese Academy of Sciences, Beijing, China

**Keywords:** alignment, sacrificial strategy, liquid metal, chip fabricating, EOP

## Abstract

This work proposes an “N2-1” sacrificial strategy to help to improve the accuracy of the bonding technique from the existing level. The target micropattern is copied N2 times, and (N2-1) of them are sacrificed to obtain the most accurate alignment. Meanwhile, a method for manufacturing auxiliary solid alignment lines on transparent materials is proposed to visualize auxiliary marks and facilitate the alignment. Though the principle and procedure of alignment are straightforward, the alignment accuracy substantially improved compared to the original method. With this technique, we have successfully fabricated a high-precision 3D electroosmotic micropump just using a conventional desktop aligner. Because of the high precision during the alignment, the flow velocity is up to 435.62 μm/s at a driven voltage of 40 V, which far exceeds the previous similar reports. Thus, we believe that it has great potential for high precision microfluidic device fabrications.

## 1 Introduction

Since the late 1990’s, microfluidics has gradually developed from Micro Electromechanical System (MEMS) technology and become an independent interdisciplinary research field (Whitesides, 2006; Fan et al., 2018). In recent years, microfluidics has been widely applied in bio (chemical) analysis (Zheng et al., 2004), drug screening (Sun et al., 2020), cell handing (Zilionis et al., 2017), organ-on-a-chip engineering (Huh et al., 2010; Cheng et al., 2017), nanomaterial preparation (Yu et al., 2017) and other specific fields. By manufacturing complex multi-channel three-dimensional structures, microfluidic chips can achieve more complex functions (Sharma et al., 2015). At present, the rapid development of direct three-dimensional (3D) fabrication technology has satisfied the needs of many stereoscopic microfluidics chips (Bhattacharjee et al., 2016; Sochol et al., 2018). And there are researches using 3D printing unique polymer molds instead of soft lithography to build multi-layer non-planar polydimethylsiloxane (PDMS) to achieve integrated microfluidics system (Glick et al., 2016). But this method is currently constrained by factors such as specific materials and complex design. Therefore, assembling the chips face to face is a cheaper, faster and also more general technique.

A critical requirement in the development of 3D multilayer-based microfluidics chips fabrication is the accurate alignment (Anderson et al., 2000), which is seen as moderately challenging work (Kipper et al., 2017). An accurate chip assembly technology could expand its potential for applications, especially in improving detection performance (Chuang et al., 2021; Wang et al., 2021) and commercialization (Kumar et al., 2013). Especially for the alignment of the plasma bonding process, two pieces should be bonded as soon as possible after being treated by plasma. Normally only a very short time is allowed for the alignment before bonding, which makes the alignment even harder.

To successfully assemble the chips, a variety of ways have been attempted. One and the simplest method is to align by manual observation and manipulation. However, large alignment offset and non-repeatability make it suboptimal for integrated microfluidic chips requiring high alignment accuracy (Kim et al., 2005). In this case, approaches to align according to the concave-convex pair structures on the chips have been reported (Yoon et al., 2012; Wu et al., 2018). By adjusting the position and pressing by hand, the alignment of the two layers of chips can be achieved. But manual alignment accuracy is still hard to improve due to the individual skill dependency, properties of chips materials, and other uncontrollable factors.

To avoid unreliable manual alignment, mechanical aligners or alignment techniques have been set up to improve precision (Jeong et al., 2009; Li et al., 2015). Conventional mask aligners require several manual steps for optical alignment in a short time. And the limited field of view causes inconvenience to operate under the microscope. Cottet et al. (2017) added a custom-made steel chuck for the mask aligner to precisely control PDMS slab. Another approach is based on attaching PDMS to the attachment of the microscope objective, which can achieve accurate and reliable alignment (Sivakumarasamy et al., 2014; Punniyakoti et al., 2017). And an alignment system to avoid the post-misalignment problem caused by the transferring process is investigated (Wang et al., 2018). In most cases, mechanical aligners usually affect the bonding strength of the chip due to the long operation time between the plasma treatment and final bonding. And the existence of the optical system also increases the cost. Moreover, the accuracy obtained using the alignment mechanicals still heavily depends on the operators’ skill, and even the most experienced technicians can produce errors of tens of microns during the assembly process.

Therefore, some fast, straightforward chip-level alignment techniques have been developed. Ding et al. (2011) proposed a capillary-driven automatic packaging (CAP) technique. The technique utilized structurally directed capillary inter-actions to establish self-alignment and self-engagement in an automatic process. Lu et al. (2012) reported a magnet-assisted device-level alignment to fabricate membrane-sandwiched PDMS microfluidic chips. In this method, pairs of magnets are placed symmetrically on each PDMS layer, and the magnetic attraction will automatically pull the PDMS layer to an aligned position during the assembly process. Mou et al. (2019) developed a hinge-based aligner for the assembly of microfluidic chips. The two chip-holders are rotated and aligned in a hinged manner to achieve chips alignment. However, these aligners are often limited by the additional microfabrication processes or the characteristics of the chips’ materials.

Furthermore, in order to prevent instant adhesion after plasma treatment, during the PDMS stacking and layer alignment process, lubricants such as ethanol and methanol are used to promote smooth movement between the layers to facilitate alignment adjustment (Hosokawa and Maeda, 2000; Mogi and Fujii, 2013). Both manual alignment (Mogi and Fujii, 2010) and under-microscope adjustment (Byung-Ho Jo et al., 2000) can be combined. However, this method may introduce shear force at the solid-liquid interface in the bonding process, causing a certain alignment error (Niklaus et al., 2003). Therefore, it is worth exploring an alignment method with convenient application, broad applicability, and improved alignment accuracy.

In this paper, a technique applied in the face-to-face chip bonding to realize high precision alignment of individual micropatterns is presented. This work proposes an “N^2^-1” sacrificial strategy to help to improve the accuracy of the bonding technique by one order of magnitude from the current level. For example, if the micropattern in the center of the matrix is designed to be perfect aligned, while the micropattern next to it is designed to be shifted to the left by 5 μm. When the bonding is shifted to the left by 5 μm, although the micropattern in the center is not perfectly aligned, the one next to it will be perfectly aligned instead. Under this sacrificial strategy, within a specific range of alignment accuracy, one of the N^2^ micropatterns will always be perfectly aligned with all the others sacrificed. Chips using this technique are not limited by factors such as material and thickness. We use Python 3.8 to calculate the alignment probabilities of this method for different offsets. The practicability and regularity of the method have also been proved. For chips fabricated by transparent polymer such as PDMS, we add liquid metal alignment guidelines to reduce alignment difficulty. Without any auxiliary instruments, the chips with this technique can be quickly aligned. Experiments show that this method can achieve a higher alignment accuracy in a short period of bonding time. Moreover, the technique can be combined with other alignment techniques or desktop aligners for even higher accuracy. We have achieved a high-precision alignment of 3D electroosmotic micropump electrodes and increased the flow rate.

## 2 Design and methods

### 2.1 Error-tolerant matrix

#### 2.1.1 Chip design principles

The technique depends on the chip design. In this sacrificial strategy, the target micropattern is copied N^2^ times, and (N^2^-1) of them are sacrificed to obtain the most accurate alignment. The details of this strategy are as follows: The N^2^ micropatterns are arranged in an N × N matrix on both sides of the bonding layers. While only the micropattern in the center of the N × N matrix is designed to be perfectly aligned. All the other micropatterns are designed to shift a little bit away from their original perfect alignment position based on their distance from the center. The further the position from the center, the larger the distance would be shifted. Thus, when the error of the alignment happened, although the center micropattern was not aligned perfectly, the micropattern at a certain position will be perfectly aligned by chance. Under this sacrificial strategy, within a specific range of alignment accuracy, one of the N^2^ micropatterns will always be perfectly aligned with all the others sacrificed.

**FIGURE 1:**
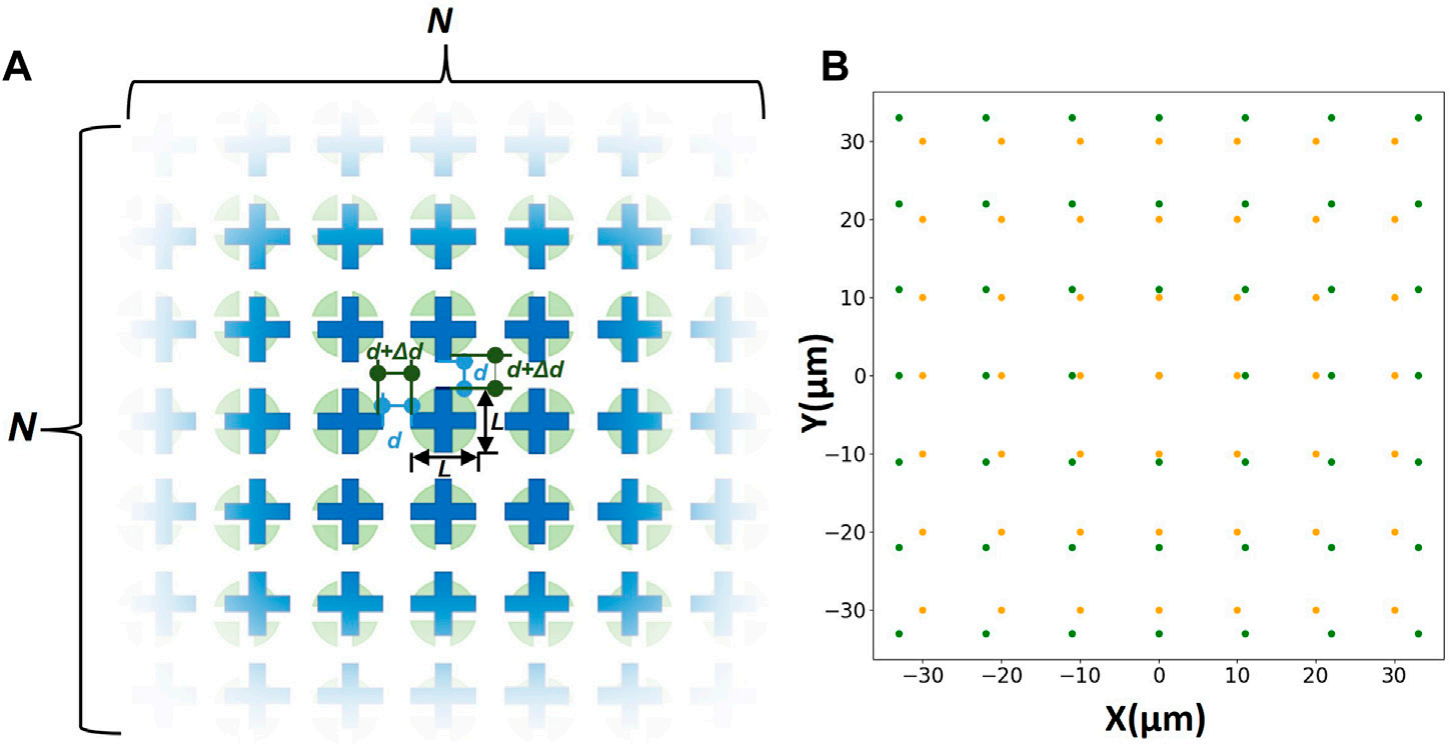
**(A)** Schematic diagram of the two-layers matrix structure. The blue layer with N x N crisscross micropatterns is the upper layer, and the green layer with N x N circles is the lower layer. **(B)** The coordinate diagram of the matrix (N = 7 μm, L = 5 μm, d = 5 μm, Δd = 1 µm).

Based on this principle, micropatterns of two layers are designed separately. First, the micropatterns in both layers are arranged as a matrix of N × N. As shown in Figure 1A, the size of micropatterns is L μm. Meanwhile, a certain spacing d μm is maintained between every two adjacent micropatterns in one of the layers. And d + Δd µm in the other. Δd µm is the shift distance. In this way, even if the central micropatterns in the matrix of two layers cannot be precisely aligned, another pair of structures in the matrix still can remedy errors and achieve alignment due to the difference in spacing of the two layers’ micropatterns.

In order to facilitate subsequent calculations, the coordinates are drawn using Python 3.8, and a certain transformation is performed to make the center micropattern can be located at the origin of the coordinate (Figure 1B). And we number each micropattern of the matrix as follows:

$$\left\lceil \begin{array}{ccccc} \cdots & \vdots & \vdots & \vdots & \cdots \\ \cdots & \left(-i,+j\right) & \left(0,+j\right) & \left(+i,+j\right) & \cdots \\ \cdots & \left(-i,0\right) & \left(0,0\right) & \left(+i,0\right) & \cdots \\ \cdots & \left(-i,-j\right) & \left(0,-j\right) & \left(+i,-j\right) & \cdots \\ \cdots & \vdots & \vdots & \vdots & \cdots \end{array}\right\rceil$$ (1)

$$\begin{array}{c} i\leq \frac{N-1}{2},i=1,2,3\cdots \\ j\leq \frac{N-1}{2},j=1,2,3\cdots \end{array}$$

#### 2.1.2 XY offset and the technique accuracy

The offset of the entire matrix, namely, the central offset, is (X, Y). X is the horizontal offset, and Y is the vertical offset. They are determined by,

$$\left\{\begin{array}{c} X=\pm i∆d\\ Y=\pm j∆d \end{array}\right.$$ (2)

**FIGURE 2:**
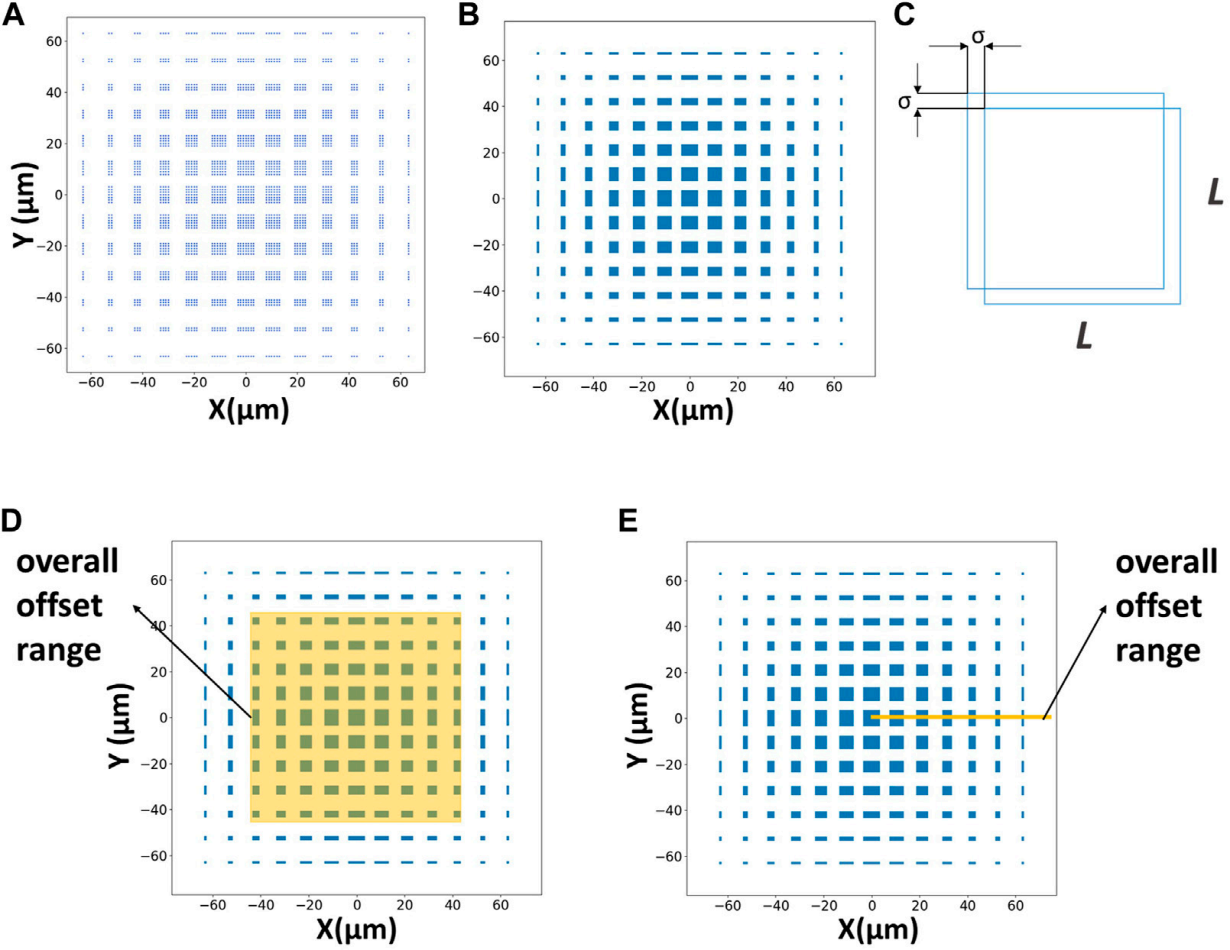
Measurements of alignment offsets. **(A)** The blue area represents the offset of the matrix when there is a pair of totally aligned micropatterns (N = 7 μm, L = 5 μm, d = 5 μm, Δd = 1 µm). **(B)** The blue area represents the offset of the matrix when there is a pair of aligned micropatterns with 0.5 µm alignment accuracy (N = 7 μm, L = 5 μm, d = 5 μm, Δd = 1 μm, σ = 0.5 µm). **(C)** The alignment accuracy measurement. In order to facilitate the calculation, only consider the XY offset, and both the accuracy are set to σ. **(D)** Schematic diagram of the area method. **(E)** Schematic diagram of the length method.

As we known, the top and bottom layers each have N^2^ micropatterns. Although the patterns are designed to align with each other center-to-center, in fact, when any micropattern in the top layer is perfectly aligned with any micropattern in the bottom layer, the alignment can be regarded as a successful alignment. So, theoretically, each micropattern in the top layer has N^2^ chances in the bottom layer to be perfectly aligned with. Then there will be N^4^ kinds of perfect alignment cases under this technique. The corresponding deviation is (X, Y). Every situation will sacrifice the other N^2^-1 pairs of micropatterns in the matrix. In this way, using Python to program and calculate these cases, we can intuitively see the alignment situation. We can work out the offset of the matrix when there is a pair of totally aligned micropatterns (Figure 2A). However, when the experimental micropatterns allow a certain little bit of error, which is called σ, this method will achieve a higher probability of successful alignment (Figure 2B). As the figure shows, the alignment area has changed from discrete points to squares. And the maximum allowable offset σ can be regarded as the alignment accuracy of the technique under this kind of parameter layout (Figure 2C). For example, when σ = 5 µm is specified, the alignment error below 5 µm in this method can be regarded as perfect alignment. At this time, the alignment accuracy is 5 µm. Therefore, a certain parametric study is required.

#### 2.1.3 Probability calculation of successful alignment

According to the previous section, the blue area of the calculated graphics indicates that there is a pair of micropatterns to achieve alignment under the corresponding offset, and the white area indicates that there is no micropattern to achieve perfect alignment. Therefore, we can calculate the probability of successful alignment in different overall offset ranges and under different parameters by programming independent repeated experiments.

First, the probability of successful alignment is calculated based on the area ratio of the successful alignment area in the image to the overall offset range (Figure 2D). This method is intuitive and conforms to the actual logic. But this method is too time-consuming for calculation and drawing graphics. This method greatly affects the calculation efficiency and is restricted by the accuracy of the drawing. To reduce the computational cost, we adopt a simplified length method as shown in Figure 2E to estimate the probability. Taking the image center as the starting point, we draw a line segment representing the range of the overall offset and calculate the probability of successful alignment in the X direction by the ratio of the length through the successful blue area to the total length. Since the figure maintains symmetry during the design of the structure and experiment, this method can be applied to the entire matrix. This method not only meets the requirement of accuracy but also greatly improves calculation efficiency.

### 2.2 Liquid metal auxiliary alignment marks

The structures’ shape on chips made of transparent materials such as PDMS is usually difficult to distinguish with naked eyes. Therefore, converting the transparent alignment auxiliary marks into opaque is beneficial for improving the alignment accuracy. Moreover, with the help of liquid metal auxiliary line, the angular tilt can be avoided. Small angular tilt can be easily recognized by extending the length of the auxiliary line.

**FIGURE 3:**
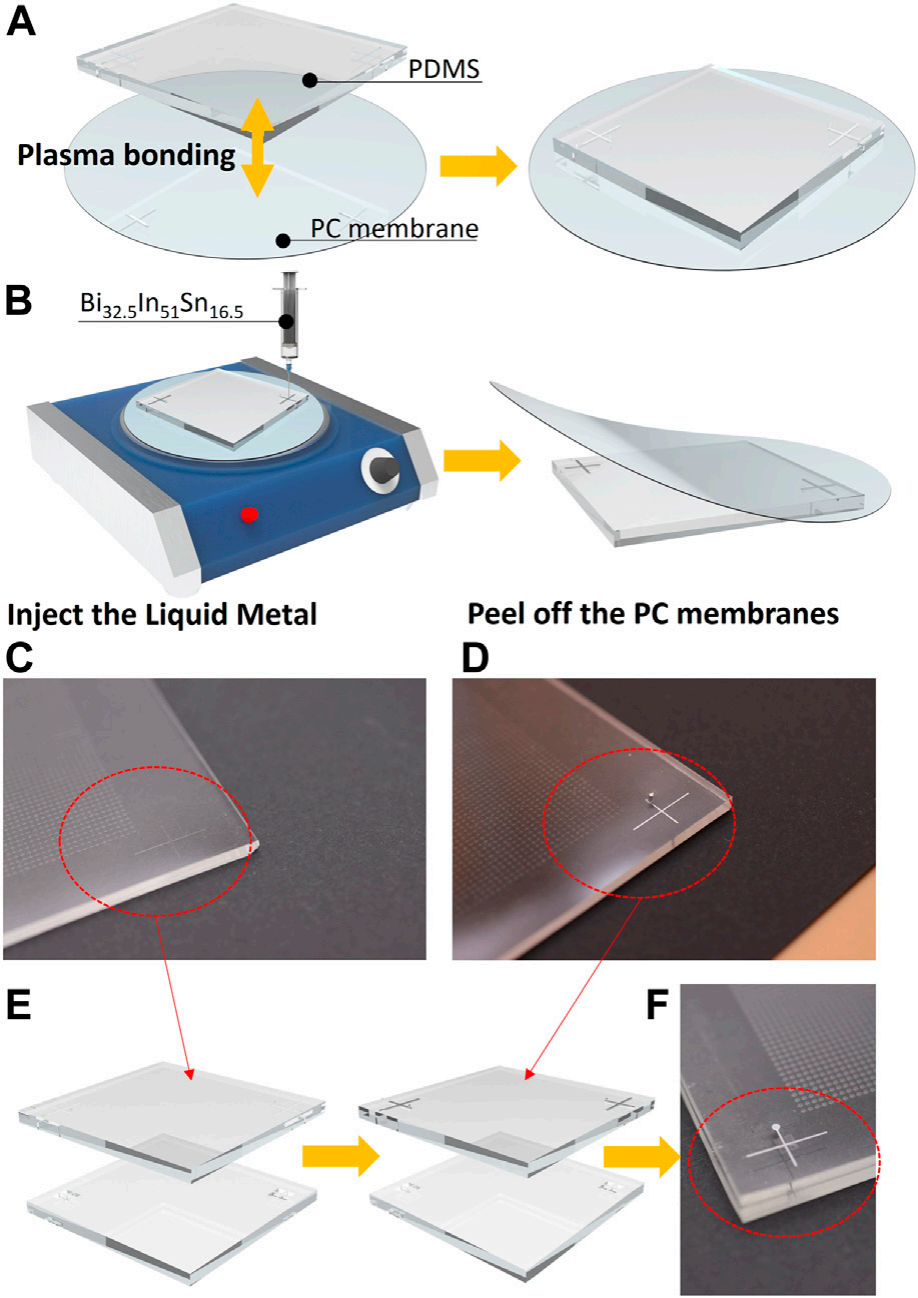
**(A)** Brief process of plasma bonding the PC membrane with the PDMS chip. **(B)** Brief process of producing liquid metal guidelines. **(C)** and **(D)** Comparison of liquid metal auxiliary wire before and after production. **(E)** Diagram of fabrication process and alignment process of upper and lower liquid metal wires. **(F)** Local top view of the auxiliary liquid metal lines.

The operation method based on reversible bonding technology is described below as shown in Figure 3. (Hong et al., 2021). First, a 30 μm high mold is made of negative photoresist SU-8 2050 (MicroChem Corp., Westborough, MA, United State). The mold consists of the Error-tolerant structure matrix and microchannels for auxiliary marks which are distributed in four corners. Then, the PDMS (A: B ratio of 8: 1 by weight; Dow Corning, Midland, MI, United State) is poured onto the mold and baked at 65 C for 2.5 h. After peeling off the PDMS chip and making holes on the auxiliary channels with a puncher, the PDMS chip is bonded with a PC membrane (Whatman, Part of GE Healthcare, United State). After those steps, Bi_32.5_In_51_Sn_16.5_ (60 C) is injected into the microchannels for marks (To prevent the liquid metal from condensing after entering the microchannel, the operation is carried out on a hot plate). Finally, when the liquid metal is cooled down to room temperature after removing chips from the hotplate, peeling off the PC membrane, the liquid metal marks are formed on the chips and ready for alignment. Then, the chips can be bonded face to face after oxygen plasma surface treatment (Supplementary Movie S1).

## 3 Results and discussion

### 3.1 The effect of N on the probability of successful alignment

**FIGURE 4:**
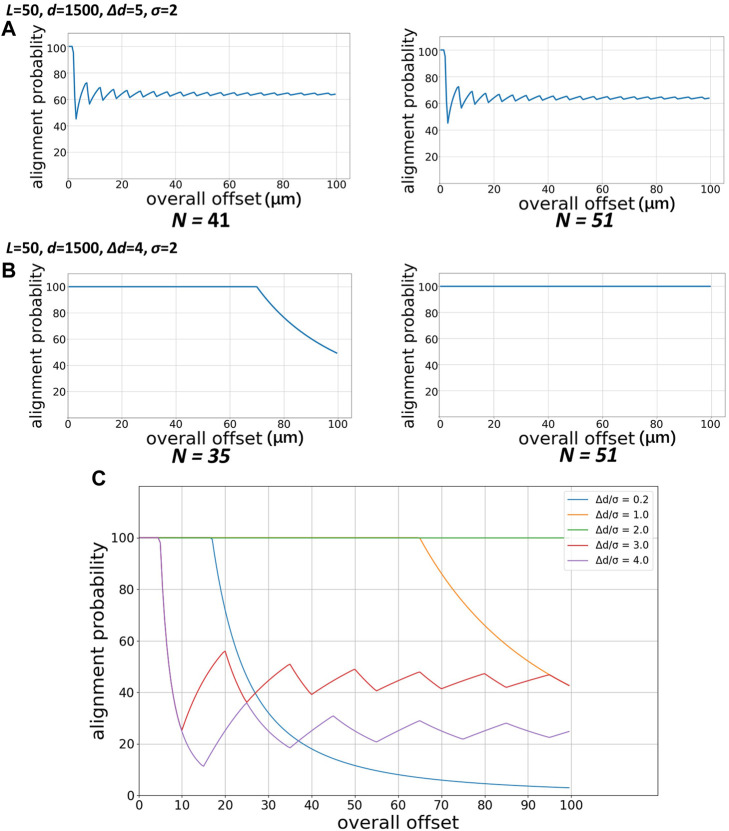
Probability of successful alignment varied with overall offset with different Δd and different N. **(A)** Δd = 5 µm **(B)** Δd = 4 µm **(C)** Probability of successful alignment of a matrix with the same parameters under different Δd arrangements.

Figure 4 illustrates the probability of successful alignment with different N. As we can see, when N becomes larger with other parameters unchanged, there is no noticeable difference in the probability of successful alignment (Figure 4A). As shown in Figure 4B, the larger N will help to increase the “safe range” of the overall offset, in which the alignment probability is 100% and perfect alignment will occur for sure. At the same time, to evaluate the performance of the technique in a more targeted manner, we set the upper limit of the overall offset to 100 µm during the parametric study. Therefore, when using this method, it is necessary to properly design the matrix and select N according to the experimental requirements and the normal error range of alignment.

### 3.2 The effect of Δd on the probability of successful alignment

In the numerical study, we find that the ratio of the shift distance Δd to the maximum allowable offset σ in the matrix parameters has an essential influence on the probability of successful alignment of the technique. Figure 4C shows the calculation of the impact of Δd under the conditions of N = 25, L = 5000 μm, d = 1500 μm, and σ = 5 µm. When Δd/σ = 2, this technique can maintain 100% probability of successful alignment within a large overall offset range. When Δd/σ < 2, the 100% probability of success requirement can still be met within a certain overall offset range, successful alignment cannot be guaranteed when the offset is too large but the probability goes smoothly down all the time; when Δd/σ > 2, the shift distance cannot compensate for part of the alignment offset, so the probability decreases rapidly and began to fluctuate afterwards.

### 3.3 Significance of “N^2^-1” alignment for microfluidic chip production

**FIGURE 5:**
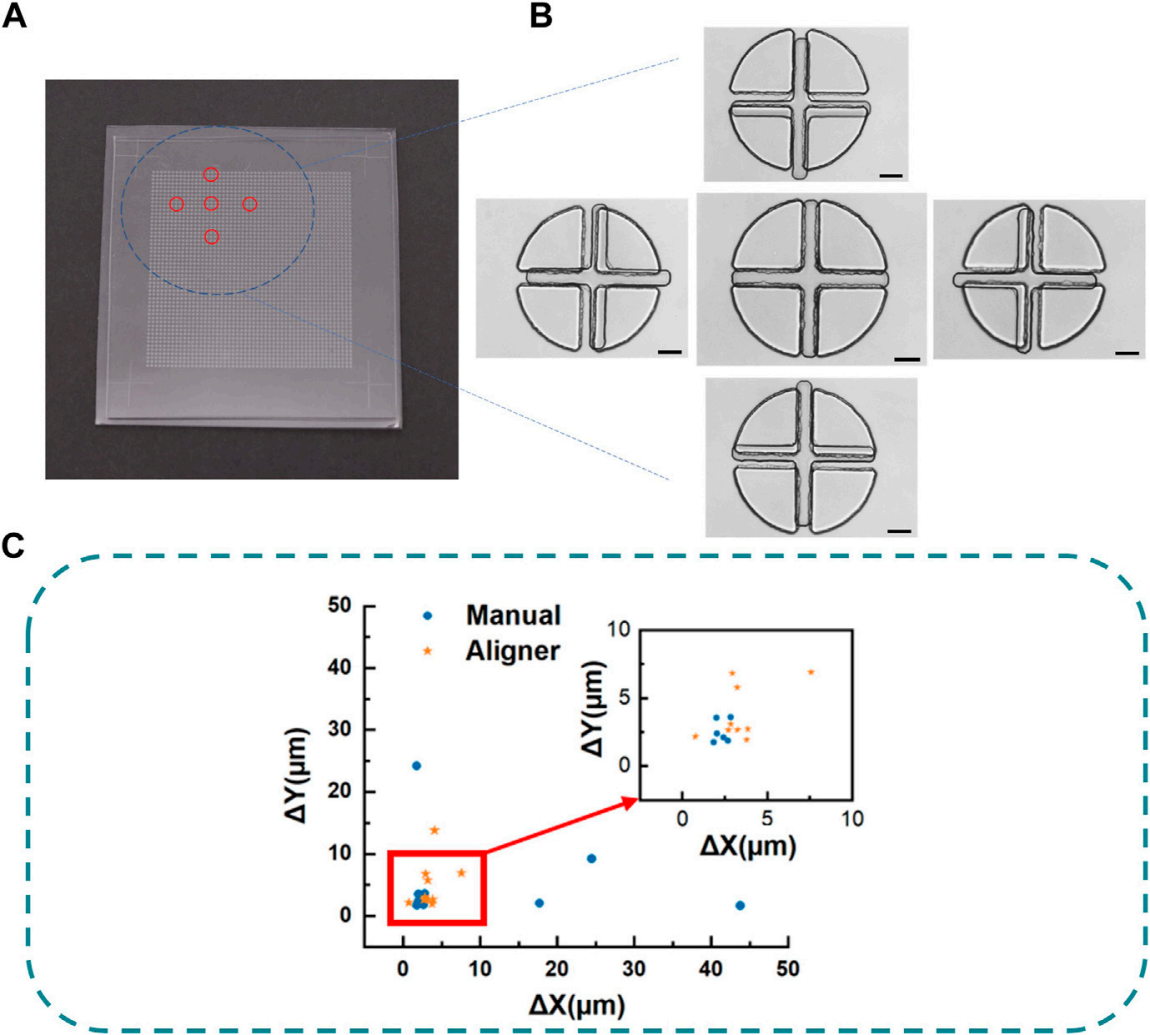
**(A)** Picture of the bonded PDMS chip. **(B)** Microscope image of the perfectly aligned micropattern in contrast to other surrounding micropatterns in the N^2^ micropatterns matrix. Scalebar is 100 µm. **(C)** Alignment error of manual and aligner based on sacrificial strategy.

For illustration and testing, we apply this “N^2^-1” sacrificial strategy and the liquid metal-based auxiliary mark technique to fabricate a PDMS chip. As shown in Figure 5A, both PDMS layers have a 51 × 51 micropatterns matrix (One is a matrix of cross, and the other is a matrix of circle-like shape). The target is to align the center of a cross pattern with the center of a circle during a quick face-to-face bonding with a maximum allowable error of only 2 µm. The distance between two adjacent crosses is 300 μm, and that of the four-petal circle is 302 µm (N = 51 μm, L = 600 μm, d = 300 μm, Δd = 2 µm). With the help of auxiliary marks at the corners, both PDMS slab was easily preliminarily aligned and quickly bonded face to face by naked eyes in only several seconds after plasma treatment. The high-precision alignment of a pair of micropatterns is achieved with an alignment accuracy of 1.633 μm (Figure 5B). To discuss the statistical results of this strategy, we repeated the alignment bonding tests ten times separately for manual alignment. And we also tested the aligner-based alignment accuracy (WH-AM-01, Wenhao Co., Suzhou, China), to prove that the sacrificial technique also could further improve on other alignment methods. As shown in (Figure 5C), if we aligned the bonding by naked eyes with our technique, all the alignment error is below 50 μm and 60% of the alignment error was below 5 μm. The average differences were (10.166 ± 14.275 μm, 5.235 ± 7.045 μm). While, if we use an aligner, the average differences were (3.510 ± 1.702 μm, 4.844 ± 3.684 μm). These show that our technique can greatly improve the alignment accuracy of the original method.

### 3.4 3D electroosmotic flow pump (EOP) fabrication with high precision alignment

**FIGURE 6:**
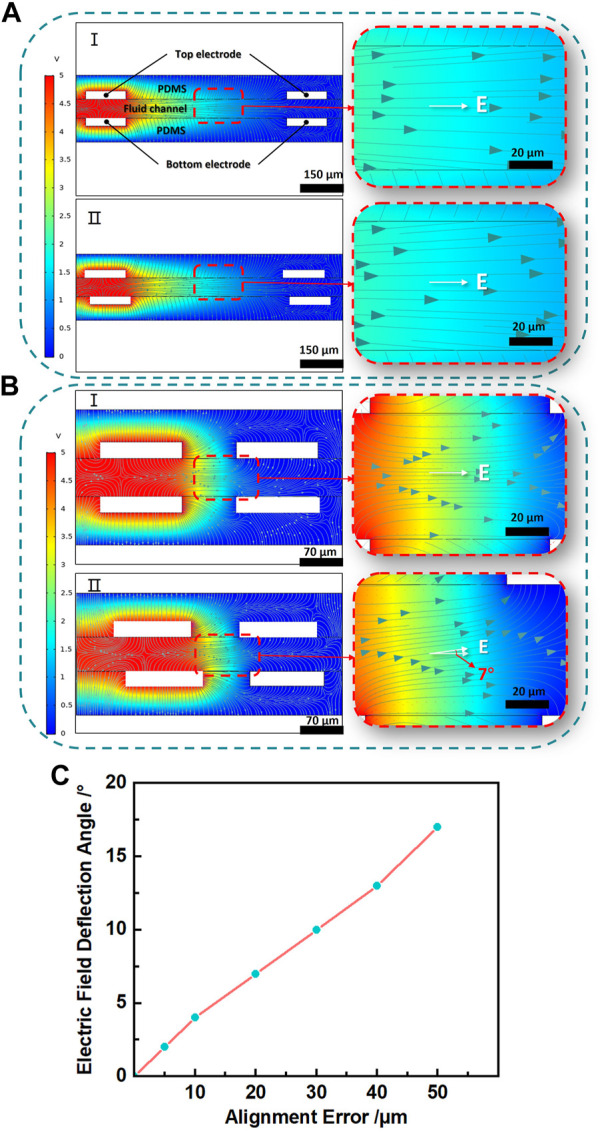
**(A)** I. Electric potential and electric field simulation when the top and bottom electrodes are completely aligned with wide electrode spacing. II. Simulation when the top and bottom electrodes with misalignment and wide electrode spacing. **(B)** I. Simulation when the top and bottom electrodes are completely aligned with narrow electrode spacing. II. Simulation when the top and bottom electrodes with misalignment and narrow electrode spacing. The right image is the enlarged partial image of the left image, and E is the electric field direction in this area. **(C)** Relationship between alignment error and electric field deflection for the smaller EOP.

As we known, liquid metal-based electroosmotic micropump with vertically placed electrodes requires bonding of the upper and lower electrodes to relatively high accuracy in a short time especially when the EOP is only hundreds of microns. As a demonstration, Figure 6 shows how the alignment accuracy affects the electric field generated in an EOP. With an alignment error of 20 μm, for the large EOP (Figure 6A, electrode distance of 600 µm), the electric field direction is not affected too much. But for the smaller EOP (Figure 6B, electrode distance of 100 µm), with the same alignment error, the electric field direction is obviously deflected by about 7°.

Figure 6C shows how the alignment error affects the electric field direction in a small EOP. As the alignment error increases from 5 µm to 50 μm, the deflection angle of the electric field direction expands approximately linearly from 2° to 17°, which means the alignment error is better to be controlled below 5 μm for the small EOP. The simulation was performed with commercial software Comsol 5.5 (COMSOL Inc., Stockholm, Sweden).

In the early attempt, researchers placed the liquid metal electrodes vertically and put multiple microchannels parallelly in the middle to increase the flow rate (Gao and Gui, 2014; Fu and Gagnon, 2015; Gao and Gui, 2016; Ye et al., 2019). Because the alignment accuracy was not high enough in their work and there was an alignment error of tens of microns (Ye et al., 2019), they set the electrode spacing as long as 8 mm to make sure the electric field direction inside the flow to be parallel to the microchannel.

**FIGURE 7:**
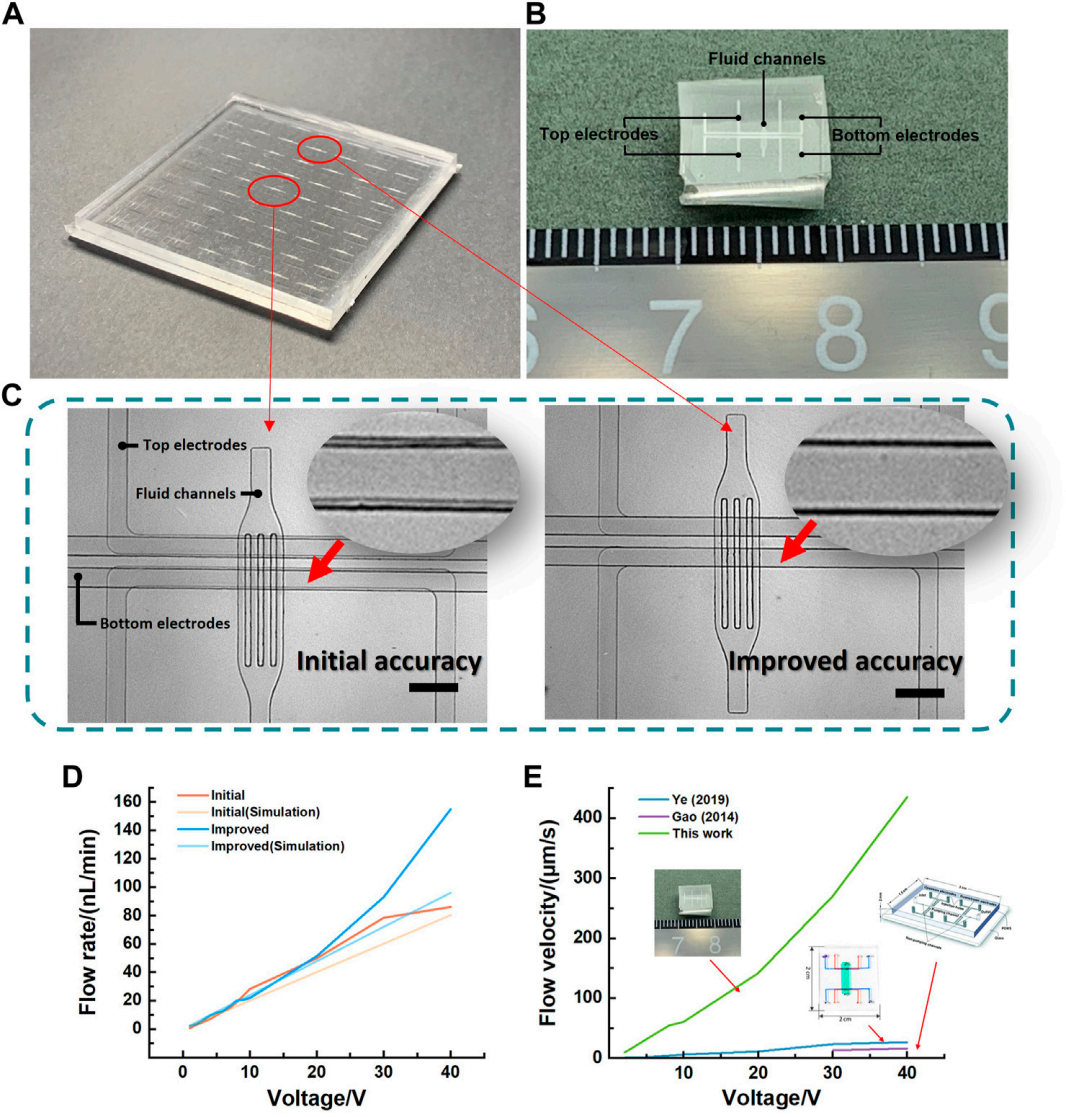
**(A)** Picture of the bonded 3D micropump chip including two electrode layers and a fluid layer with 7 × 9 micropatterns on each layer. **(B)** Comparison of central pump and optimally aligned pump. Scalebar is 300 µm. **(C)** Optical photograph of the separated micropump chip after alignment. **(D)** Comparison of flow rates from 1 V to 40 V for the optimally improved micropump and the initial micropump and their simulation results. **(E)** Comparison of this work with Gao’s (Gao and Gui, 2014) and Ye’s work (Ye et al., 2019) with similar dimensions.

To minimize the alignment error and shrink the size of EOP, the sacrificial strategy was performed during the fabrication in this work. A traditional desktop aligner was used for initial bonding alignment. As shown in Figure 7A, a matrix of 7 × 9 micropatterns was applied for the sacrificial strategy to align the electrode layers. A little different from the former analysis, in this EOP design, because the electrodes alignment accuracy was only required in the direction parallel to the flow channel, we only designed the shift distance Δd in that direction but still put all the micropatterns in a matrix manner. Because there were 63 micropatterns in total, the alignment accuracy was enhanced 63 times higher during the process of bonding. The structure design of the micropump was basically based on Ye’s work (Ye et al., 2019). Particularly, we reduced the electrode spacing from 8 mm to 100 μm and fluid channels from 116 μm to 50 μm. Details of the chip preparation were given in the Supplementary Figure S2. And the flow rate was measured by the particle tracer method (Polystyrene microspheres, Aladdin, China). As shown in Figure 7B, the alignment offset was improved from initial 24.217 μm–3.415 μm. The best aligned micropump and initial micropump were then separated (Figure 7C), fabricated and tested. Figure 7D shows the flow rate with the driving voltage from 1 V to 40 V. The flow rate of best aligned micropump was 1.88 nL/min at 1 V, and 96.02 nL/min at 40 V. The best aligned pump has a more stable and slightly higher flow rate than the initial pump due to perfect alignment and low electric field deflection. This coincided with the simulation where the improved micropump flow rate was slightly larger than the initial micropump flow rate. Moreover, the lowest driving voltage of improved micropump was only 1 V, which is much lower than the former work. And with similar design parameters and materials, the flow velocity at 40 V of Ye’s pump was 27.52 μm/s, and Gao’s pump was 16.57 μm/s. In this work, the flow velocity reached 435.62 μm/s at 40 V. Also, this micropump was smaller in size (Figure 7E). This improvement was due to the increased electric field strength by reducing the electrode spacing to 100 μm with higher alignment accuracy.

In addition, this technique can be extensively combined with other alignment techniques or desktop aligners to further improve the alignment accuracy.

## 4 Conclusion

In this paper, we have developed a precision enhanced alignment bonding technique with an “N^2^-1” sacrificial strategy. Compared with other alignment devices or methods, the “N^2^-1” technique has several unique features, including a) improving alignment accuracy from any existing accuracy level; b) the feasibility of specific design for different micropattern matrices; c) compatibility with other aligning techniques or desktop aligners; and d) stable bonding result irrelevant to operators’ skills. Through parametric research, we gave the optimal value of Δd/σ that can guarantee the probability of successful alignment within a certain overall chip offset. Thus, principles have been provided for matrix design. For transparent materials such as PDMS, we proposed a method to draw liquid metal auxiliary marks on the bonding face for easier bonding alignment. The technique we proposed is novel and handy. With help of this technique, we achieved a bonding accuracy as high as 5 µm for a 300 µm micropattern. Then we successfully shrank the electrode space from 8 mm to 100 μm for a 3D electroosmotic micropump with an alignment error of only 3.415 μm. A fluid flow velocity of 435.62 μm/s was achieved at 40 V, and the lowest driving voltage was 1 V. Although the “N^2^-1” technique requires huge material sacrifice to achieve precise alignment, however, it is worthwhile to make certain sacrifices for some high precision alignment of small structures, because without this technique the required structure may be impossible to be fabricated. It depends on how important the structure is. Moreover, the sacrificial material is only PDMS which is relatively cheap. The silicon wafers and photoresist used as templates can be reused for the next fabrication. So, we believe that it has great potential for high precision microfluidic device fabrications.

## Data Availability

The original contributions presented in the study are included in the article/Supplementary Material, further inquiries can be directed to the corresponding author
