# Supplementary material for: Precision enhanced alignment bonding technique with sacrificial strategy
Source: Front Bioeng Biotechnol. 2023 Feb 16;11:1105154. doi: 10.3389/fbioe.2023.1105154 (PMC9978516; doi:10.3389/fbioe.2023.1105154)
Supplement: Supplementary file 1 [file Table1.DOCX]

Supplementary Material

## Supplementary Figures


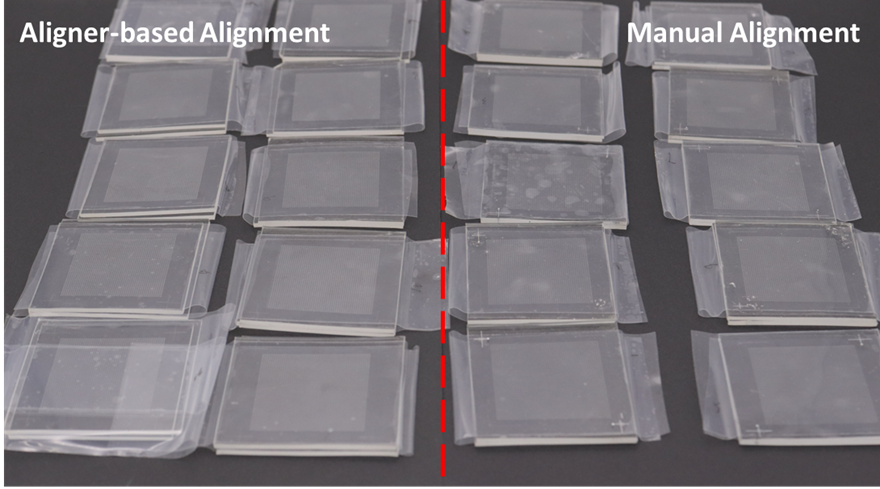


**Supplementary Figure 1.** The aligner-based alignment and manual alignment accuracy tests.

**
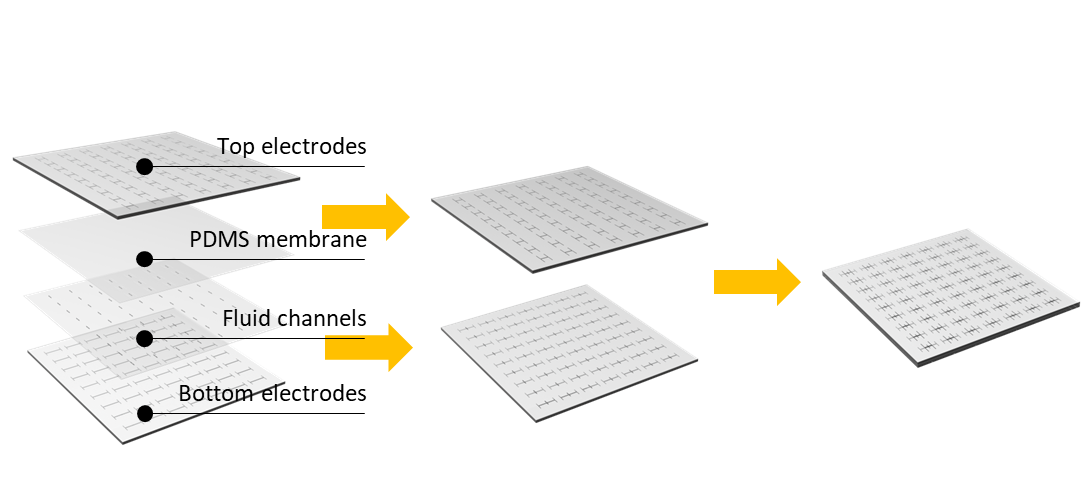
**

**Supplementary Figure 2.** Schematic diagram of EOP bonding sequence

The EOP consisted of four layers: the top and bottom electrodes layers, the fluid channels layer and a blank PDMS membrane sealing layer. Layers were manufactured by standard soft lithography and then directly pouring or spin coating PDMS on patterned silicon wafers.

First, we bond the thick bottom electrodes layer to the fluid channels layer (1500 RPM, 42 µm) and remove the two layers together. Then combined the top electrodes layer with a blank PDMS membrane (3000 RPM, 15 µm). In this way, two parts of the structure are formed, each with two layers.

The second step is to align and bond the two parts. In this step, for precise control of the top and bottom electrode positions, an aligner (Wenhao, Suzhou, China) was used to do the alignment. Two PDMS layers were stuck on two platforms of the aligner respectively. We selected the matrix center structure or the relatively perfect structure for initial alignment and made the alignment under a microscope. After bonding the four-layer structure, the best aligned micropump and initial micropump were then separated. Inlets and outlets of electrodes and fluids were punched with a 1.2 mm hole puncher.The gallium-based alloy was injected into the electrode channels to form electrodes. Copper lines were used to connect electrodes with a power source. Package adhesive sealant is used to seal the copper wire and liquid metal.
